# Fragment analysis in forensic anthropology

**DOI:** 10.1080/20961790.2020.1811513

**Published:** 2020-12-28

**Authors:** Douglas H. Ubelaker, Yaohan Wu

**Affiliations:** National Museum of Natural History, Smithsonian Institution, Washington, DC, USA

**Keywords:** Forensic sciences, forensic anthropology, fragments, radiocarbon

## Abstract

Anthropological analysis of fragmentary evidence can be challenging but diverse methods allow substantial information to be gleaned. Scanning electron microscopy/energy dispersive X-ray spectroscopy enables determination if bone and/or tooth tissue is present. Protein radioimmunoassay or DNA analysis can establish the species present. Histological analysis can assist in species determination and reveal information about thermal changes. Radiocarbon analysis with special reference to the modern bomb-curve can clarify the postmortem interval. Anthropologists should also be aware that DNA analysis not only can enable positive identification but assist in the evaluation of sex and age at death.

## Introduction

With increasing frequency, forensic anthropologists are requested to report on fragmentary remains. Criminal activity and taphonomic factors can produce skeletal fragmentation. Blast trauma, gunshot injury and blunt force trauma all can shatter bones and produce fragmentary evidence. Postmortem events involving fire, weathering, animal scavenging and trampling usually fragment bones as well. Crime scene investigators realize that through molecular analysis, recovery of fragmentary evidence potentially can lead to positive identification. These factors combine to increase the demand for anthropological analysis of fragmentary evidence.

Clearly, analysis of fragmentary evidence is not ideal and can prove challenging for even the most experienced forensic anthropologists. However, recent research indicates that much can be learned from such evidence. Analysis can focus on a variety of issues that collectively may lead to positive identification (usually through molecular analysis) and assist in evaluating evidence for foul play. This article surveys the existing scientific literature to clarify what methods are most applicable and what issues may be addressed.

## Bone or tooth?

Analysis of fragmentary evidence must begin with an assessment of what particles represent bone or tooth. In the aftermath of a structure fire or blast event, many particles can resemble bone or tooth. Since such evidence is almost always not in pristine condition, superficial examination may not be conclusive. Many fragments of building materials can resemble bone. Conversely, fragments of bone may resemble other materials and not be recognizable through morphology alone. Analysis should commence with visual examination aided by magnification as needed. If diagnosis is not possible with visual examination, analysis can proceed to more complex methods.

Scanning electron microscopy/energy dispersive X-ray spectroscopy (SEM/EDS) represents a tool that may be available in forensic laboratories. SEM/EDS can clarify if bone or tooth is present. SEM/EDS analysis of a submitted unknown specimen produces an X-ray spectrum that reveals the composition content. The spectrum indicates what elements are present and their relative abundance. A visual image is also provided that may prove useful to identify structural components of bone or tooth.

Through spectra analysis, SEM/EDS offers an elemental analysis approach to identify the specimen. Bone and tooth tissues contain high levels of calcium and phosphorus and relatively low levels of other elements. The calcium/phosphorus ratio in bone and tooth displays minimal variation and contrasts with elemental values of other materials recovered from forensic contexts likely to be confused with bone or tooth. In a test of this method, Ubelaker et al. [1] compared bone and tooth samples (human and non-human) from a variety of contexts (modern pristine museum materials and archeological specimens) and time periods (modern to 8 000 years ago). Values of the known bone and tooth samples were compared to an Federal Bureau of Investigation (FBI) database of many known other materials commonly recovered in forensic contexts. The method clearly distinguished the bone and tooth samples from all the other known materials. The other materials that appeared closest in structure to bone and tooth were a type of octocoral, the exoskeleton of the sea horse (a type of bone), ivory, mineral apatite, synthetic hydroxyapatite and a brand of toothpaste [1]. SEM/EDS analysis is recommended not only because it successfully can identify bone or tooth tissue, but also because the method is so minimally invasive. If analysis reveals that bone or tooth is present, enough material usually remains for additional analyses to clarify species and perhaps to obtain the molecular evidence needed for identification.

## Human or non-human?

While SEM/EDS will successfully differentiate bone and tooth tissue from other materials, it will not distinguish between human and non-human samples. All animal bone and tooth samples have similar calcium and phosphorus values. Usually in forensic casework it is important to determine species status prior to DNA analysis aimed at identification [2]. If species is not determined in advance and DNA analysis is not successful, the investigator not only fails at identification but also lacks information of the species represented. With fragmentary evidence and the destructive nature of advanced testing, investigators must thoughtfully consider the appropriate analysis sequence. Usually in medico-legal practice it is sufficient to determine if the remains are human or not. In some cases involving animal poaching or investigation of other issues related to non-human animals, it may be helpful to determine the non-human species represented.

Protein radio immuno assay (pRIA) represents a method to distinguish human samples from those of non-human animals [3]. pRIA focuses on proteins remaining in bone and tooth samples. Proteins can be retained even in ancient fossils and can be expected to be present in specimens from modern forensic cases. The technique is highly successful in recognizing human status and in determining the type of non-human animal present at the family taxonomic level. This method relies on measured antibody response that is species specific. A small amount of the unknown (about 200 mg) is separated and subsequently exposed to species-specific antisera. The extent of antigen binding reflects the most likely species and is quantified through use of a radioactive tracer (iodine-125). If the procedure indicates human status, enough sample usually remains to allow DNA testing aimed at identification [4,5]. Limitations of this technique are that few laboratories are capable of taking pRIA analysis and considerable costs are involved.

Histology offers an alternative approach to species estimation. Of course, preparation of slides for histological analysis is destructive and can limit the amount of material available for additional analyses such as DNA. Bone histology can offer useful but limited information on general species representation [6,7]. Some histological differences have been documented between human and non-human animals. The human pattern presents well-known structures such as circumferential lamellar bone, primary osteons, resorption spaces, secondary osteons and fragments of secondary osteons. In humans, primates and some other mammals the secondary osteons and fragments are relatively randomly distributed through the bone cortex [8].

In fast growing ungulates and some other non-human animals, the histological structures may appear in layered block-like forms termed plexiform bone. The presence of plexiform bone rules out humans and is diagnostic for non-human bone [9]. Note however that some fragments may be too small to reveal the plexiform pattern.

In other non-human mammals, the osteons may be present in bands or layers within the lamellar bone. Substantial banding pattern also is diagnostic for non-human bone, although some minor variations of it can be found in human bone, especially in infants [9].

While plexiform bone and/or the banding pattern may be diagnostic for non-human status, the human pattern is shared with some other animals, especially other primates [10]. Differences with additional non-human animals involve the size of the osteons and other structures but the size ranges are overlapping. Thus, histological analysis can be diagnostic for non-human status but not for human status. In addition, some disease conditions can produce abnormal bone histological patterns further complicating interpretation.

Bone histological patterns vary considerably throughout the skeleton [11]. The lack of knowledge of the skeletal anatomical location of small fragments represents an additional limitation. The equipment needed for histological analysis generally is available in forensic and university laboratories, but the technique is destructive and requires considerable labour.

## Age at death and sex

Information on age at death is very limited from bone fragments. The extent of compact bone in diaphyseal fragments provides some evidence of maturity. However, even this evidence is difficult to evaluate without knowledge of the anatomical area represented.

Possibly, histological analysis could provide some age information. However, histological patterns vary considerably depending on what bone is represented and what tissue within a bone is present. With fragmentary evidence, these variables usually remain unknown, greatly limiting access to age information.

Little evidence of sex can be gleaned from morphology of bone fragments. DNA offers the best approach to determining sex when it is important to do so [12]. Although not infallible, analysis of the amelogenin gene offers strong evidence of sex [13].

## Time since death

Estimation of the postmortem interval (PMI) or time since death represents an important aspect of the forensic investigation of recovered fragments. Procedures based on the extent of preservation or morphology are limited due to the many variables involved. While some histological approaches to assess the extent of diagenesis may be helpful [14], usually only very general estimates can be made. Fragments that have been burned offer additional challenges, although assessment of colour, microscopic morphology and crystalline structure may clarify if burning occurred and the maximum temperature reached in the burning process [15].

Radiocarbon analysis offers the best approach to evaluate time since death. If the remains predate 1950 AD, traditional radiocarbon analysis utilizing the half-life of carbon-14 of 5 730 years can clarify the antiquity.

If the fragments are modern (more recent than 1950 AD) radiocarbon values must be evaluated in consideration of the bomb-curve [16]. Atmospheric testing of thermonuclear devices in the 1950s produced large amounts of artificial radiocarbon. Through the food chain, these high levels of radiocarbon became incorporated into living organisms, including humans. These atmospheric levels rapidly increased until the early 1960s and then gradually decreased following cessation of atmospheric testing. As a result, humans living after 1950 AD incorporated elevated levels of radiocarbon into their tissues, including bone and tooth.

In forensic analysis it is important to first examine the tissue present most likely to contain modern radiocarbon. If present, hair, body fluids, nail or soft tissue represent the samples of choice since due to their formation and/or remodeling they have a relatively close relationship with atmospheric/dietary levels of radiocarbon [17]. If this initial analysis proves to be modern, a second sample of a tissue with a different formation/remodeling pattern can be analyzed to enable proper placement on the bomb-curve. Since remodeling rates slow with advancing age, it is important to compensate for the estimated age at death [18].

Fragment analysis may limit the more complex interpretations outlined above. Limitation to a single sample largely allows determination if the remains are from the post-1950 modern period or in contrast, from pre-bomb-curve times. However, if dental fragments are present, analysis of enamel may elucidate the approximate birth date, if modern. Dental enamel does not remodel. Thus, the radiocarbon found in dental enamel reflects the dietary levels at the time the tooth formed. If a dental fragment is sufficiently large, it may enable analysis of samples from two distinct formation zones that would allow placement on the bomb-curve. For example, the cusps on the occlusal surface form earlier/before the base of the crown. Radiocarbon analysis of samples from these distinct crown areas would produce a contrast in results that would enable placement on the bomb-curve. If the value of the crown base is higher than the sample from the occlusal surface, tooth formation would relate to the earlier, pre-1964 period. In contrast, if the value for the crown base was lower than that of the occlusal surface, the values would indicate a formation date after 1964.

If multiple fragments are recovered and context indicates they represent one individual, analysis of different types of bone and/or tooth may allow additional interpretation using the procedures defined above. The approach taken is problem/case specific, but it is important for forensic anthropologists to be aware of the methodological possibilities. Considerable costs are involved in radiocarbon analysis and the method may not be necessary if context and other evidence provide the necessary information on date of death.

## Molecular analysis

Although DNA analysis does not fall within the subdiscipline of forensic anthropology, anthropologists and others should be aware that molecular analysis can offer important information in addition to positive identification. As noted above, analysis of the amelogenin gene may reveal the sex of the individual. Research also suggests that DNA analysis provides some information on species, ancestry and age at death.

Although molecular analyses using protein-based DNA samples can provide some information on species, ancestry, and age at death [19,20], DNA samples recovered from bones and teeth are usually used for identifying human remains by comparing DNA profiles. DNA typing developed from skeletal and dental remains provides a high level of discrimination among individuals that helps to exclude a possible relationship between dissimilar samples. If the DNA profiles are consistent enough, statistical calculations can be performed for clarifying the confidence in the match.

The generation of DNA profiles from human remains and growth in forensic databases [21,22] in the past few decades aided the rapid advance in DNA analysis. Techniques for measuring DNA variation extracted from bone and tooth fragments have moved from restriction fragment length polymorphism (RFLP) to short tandem repeat (STR). RFLP analysis utilizes radiolabeled human-specific probes. The probes detect the variable number of tandem repeats (VNTR) polymorphism within a specific region of the human genome [23]. Although genomic DNA from human bone can yield some results in forensic identification, it is often too degraded to generate useful data [24].

STRs are the most used forensic markers because they can be found present in low-quantity DNA templates and degraded DNA samples. STRs are polymorphic in that the number of times the tandemly repeated DNA sequences repeated varies highly between individuals. Because of that, they can discriminate closely related individuals [25]. Some studies are done for evaluating STR success rates in forensic identifications. Miloš et al. [26] performed STR typing from DNA extracted from different skeletal elements from former Yugoslavian mass graves. They observed that trends in DNA typing success rates are the highest with samples from the femur. Samples from intact teeth also had exhibited high success rates.

Established in 1998, the Combined DNA Index System (CODIS) national database uses 13 STR loci, which sets the basis for the forensic DNA profiling in the US [27]. Several technical advances have facilitated multiplex polymerase chain reaction (PCR) amplification. Moretti et al. [28] tested fluorescent multiples STR systems and evaluated required PCR parameter ranges in their DNA typing. Divne et al. [29] developed a pyrosequencing assay for analyzing STR markers instead of the more often used capillary electrophoresis and found the alternative to be useful.

In addition to nuclear DNA analysis, mtDNA analysis is also useful for forensic identification. The mitochondria in cells contain multiple copies of the mitochondrial genome in contrast to the cell nucleus that only contains one copy. Therefore, the mtDNA is helpful in the analysis of degraded samples. Buś et al. [30] conducted the mtDNA analysis on skeletal and dental remains from a Viking mass grave that dated to approximately AD 880–1000 to test the possible maternal kinship between individuals. They successfully obtained 15 unique and three shared mtDNA profiles and identified two possible pairs of siblings or mother-child relationships.

Since the probability of successfully obtaining a DNA result is largely dependent upon the amount of DNA recovered, DNA extraction is crucial in determining the success of forensic investigations. Although the DNA molecules are more stabilized in the hard tissues, the extraction can still be challenging. In some cases, fragmented bones and teeth provide poor quality and low quantity genetic materials [31]. There have been advances in DNA extraction and purification, with the goal of improving DNA recovery and decreasing damage and inhibitors [32–34]. Several studies compared different kinds of more frequently used extraction methods [35–37] and found that total demineralization has the highest efficiency when performing extraction from highly degraded human remains. Other investigations examined how the DNA extraction process can be optimized by newly developed commercial DNA kits with improved sensitivity [38] and other technologies such as acoustic energy [39].

Past and recent publications have also revealed methods to recover DNA from skeletal and dental remains exposed to high temperature [40]. As noted above, DNA extraction is highly destructive. This could raise the risk of destroying the fragments without getting valuable information. Some studies developed alternative procedures to minimize the destruction [41,42]. In addition, advances have been made on expanding the numbers of loci that can be used in forensic investigations [43,44]. Hill et al. [44] established the characterization of 26 miniSTR loci that could help analyze degraded DNA in missing persons work in forensic science. In their study of evaluating DNA yield at increasing PMI, Mundorff and Davoren [45] suggested that instead of long cortical bones, small cancellous bones offer both more DNA and STR loci.

## Conclusion

Although anthropological analysis of fragmentary evidence is challenging, recent research indicates that much can be learned. To determine if the recovered fragments represent bone or tooth, SEM/EDS represents a useful method. The species represented can best be established through pRIA or DNA analysis, although histology can also reveal important information. The critical issue of time since death can be assessed through radiocarbon analysis, with special attention to the modern bomb-curve.

Anthropologists should also be aware that molecular analysis may provide additional vital information. DNA not only represents the primary means for positive identification but also can help establish the age at death and sex of the individual represented.

Many of the methods available for analysis are destructive. With the quantity issues presented by fragments, careful consideration is required in determining which procedures to employ. The analysis sequence of visual examination, SEM/EDS, pRIA, and then DNA analysis offers maximum information gained when fragments are small. If larger fragments are available, histology may offer supplemental and additional information. Determination of which and how many fragments to analyze is problem/case specific. Ethical issues may arise when only limited fragments are available and destructive analysis would leave little or no material to offer families if identification is successful. Anthropological analysis should be performed prior to genetical analysis and additional testing. Non-destructive analysis should be conducted prior to any destructive tests. Anthropological analysis can save time and maximize the information recovered if conducted in the correct sequence.
